# Factors Determining the Susceptibility of Fish to Effects of Human Pharmaceuticals

**DOI:** 10.1021/acs.est.2c09576

**Published:** 2023-06-08

**Authors:** Chrisna Matthee, Andrew Ross Brown, Anke Lange, Charles R. Tyler

**Affiliations:** Biosciences, University of Exeter, Exeter, Devon EX4 4QD, United Kingdom

**Keywords:** ADMET, ecotoxicology, environmental risk assessment, pharmaceuticals

## Abstract

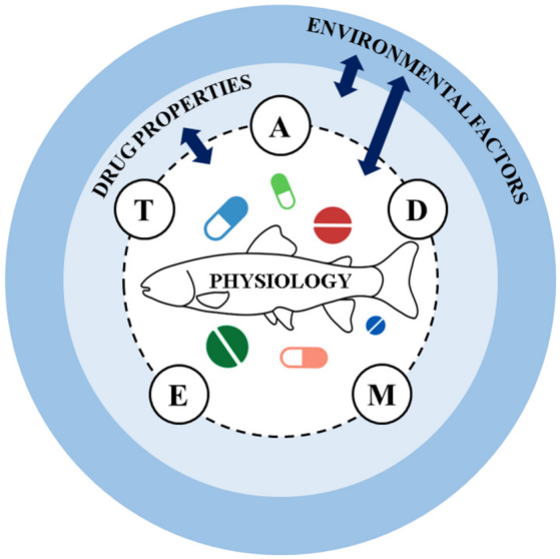

The increasing levels and frequencies at which active pharmaceutical ingredients (APIs) are being detected in the environment are of significant concern, especially considering the potential adverse effects they may have on nontarget species such as fish. With many pharmaceuticals lacking environmental risk assessments, there is a need to better define and understand the potential risks that APIs and their biotransformation products pose to fish, while still minimizing the use of experimental animals. There are both extrinsic (environment- and drug-related) and intrinsic (fish-related) factors that make fish potentially vulnerable to the effects of human drugs, but which are not necessarily captured in nonfish tests. This critical review explores these factors, particularly focusing on the distinctive physiological processes in fish that underlie drug absorption, distribution, metabolism, excretion and toxicity (ADMET). Focal points include the impact of fish life stage and species on drug absorption (A) via multiple routes; the potential implications of fish’s unique blood pH and plasma composition on the distribution (D) of drug molecules throughout the body; how fish’s endothermic nature and the varied expression and activity of drug-metabolizing enzymes in their tissues may affect drug metabolism (M); and how their distinctive physiologies may impact the relative contribution of different excretory organs to the excretion (E) of APIs and metabolites. These discussions give insight into where existing data on drug properties, pharmacokinetics and pharmacodynamics from mammalian and clinical studies may or may not help to inform on environmental risks of APIs in fish.

## Introduction

There are currently over 20,000 FDA-approved prescription drug products on the market,^1^ many of which are released into the environment daily as a result of their extensive worldwide usage as therapeutic agents. Aquatic systems are often the most significant receptors of these medicated discharges. Pharmaceuticals enter water bodies by means of multiple routes, principally through direct introduction via treated and untreated sewage (following patient use and excretion), pharmaceutical manufacturing waste streams and improper disposal of unused or expired medicines.^2−7^ Active pharmaceutical ingredients (APIs) have been detected widely including in groundwater, surface waters and wastewater treatment plant (WWTP) effluents,^8−11^ with their levels and frequencies of detection generally showing a positive correlation with the extent of their usage.^12,13^ Equally, significant levels of APIs may also develop as a result of persistent drug properties and/or inefficient sewage treatment.^4,13,14^ Recent global surveillance of 1052 sampling locations across 104 countries revealed that analgesics (29%), antidiabetics (20%) and antibiotics (15%) are the most common pharmaceutical pollutants of rivers in low to middle income countries, while antidiabetics (25%), anticonvulsants (15%) and analgesics (11%) predominate in the rivers of high-income countries.^15^ The aforementioned therapeutic classes, as well as several cardiovascular agents, antidepressants and hormones are currently detectable in the waters of all five United Nations regions, typically at levels in the low ng/L to low μg/L range.^8^ In some cases, APIs have been detected at physiologically active concentrations in wild and feral fish populations and have been causally linked to adverse reproductive, immune and behavioral effects in these organisms (see next section). Moreover, the global consumption of human pharmaceuticals is increasing owing to growing and aging populations and a general rise in chronic health conditions.^16−18^ These statistics are a significant source of environmental concern for fish, which is further compounded by the likely interactions of complex and highly dynamic API mixtures–which may have additive, synergistic or antagonistic effects–with the potential to adversely affect fish physiology and behavior.^19−23^

With many pharmaceuticals lacking environmental risk assessments (ERAs), there is a need to better define and understand the potential risks that APIs (and their biotransformation products and mixtures) may pose to fish, while also keeping the use of experimental animals to a minimum. There are both extrinsic (environment- and drug-related) and intrinsic (fish-related) factors that make fish potentially vulnerable to the effects of human drugs, but which are not necessarily captured in nonfish tests. In this review, we first briefly describe biological effects that have been observed in fish following exposure to environmentally relevant concentrations of human APIs. We then detail what is currently known about human API fate in the aquatic environment and their bioavailability to fish. Subsequently, we explore the factors that make fish, and different fish species with their distinctive physiologies and ecologies, either more or less susceptible to the exposure and effects of pharmaceuticals. We do so by framing this in relation to the absorption, distribution, metabolism, excretion and toxicity (ADMET) of APIs. It should be noted that, while the potential hazards of many APIs have previously been estimated in fish based on mammalian-derived ADME parameters,^24^ we focus on fish-specific ADMET, highlighting factors that differentiate fish from humans (and mammalian models). In these analyses, we also illustrate where existing data on drug properties, pharmacokinetics (PK) and pharmacodynamics (PD) from mammalian and clinical studies may be used to inform on environmental risks of APIs in fish, and where not, thus necessitating testing in fish (or alternatives).

## Biological Effects of APIs in Fish for Environmentally Relevant Exposure Concentrations

Most human APIs occur at relatively low exposure concentrations in the environment with a small likelihood of causing adverse effects,^4^ but their potent nature and ability to accumulate (in some cases) could lead to chronic effects via sublethal modifications to physiological processes with subsequent consequences on the behavior and fitness of wild fish.^14,25−27^ Ecological life history traits may render some species more susceptible to chemical exposure than others, as shown by the higher susceptibility of short-lived fish to the effects of endocrine active substances, compared to longer-lived species.^28,29^ Different fish life stages may also have different susceptibilities to API exposure due to, for example, life stage-specific expression of drug target proteins.^30^

Levels of human pharmaceuticals detected in aquatic environments have, for most cases, not been directly linked to immediate or long-term (chronic) adverse effects in fish. Exceptions to this include for exposure to the persistent synthetic estrogen, 17α-ethinyloestradiol, causing feminization of male fish,^31−33^ evidence for the deterioration in the general health of both rainbow trout (*Oncorhynchus mykiss*) and brown trout (*Salmo trutta*) exposed to a low level of the nonsteroidal anti-inflammatory drug (NSAID) diclofenac,^34,35^ and an indication for masculinization of female fish for exceptional cases of environmental exposure to the antifungal azole, clotrimazole.^36^

It is also increasingly being recognized that many neuroactive pharmaceuticals, even at low, environmentally relevant concentrations, can accumulate in fish brain tissues, causing alterations in neurotransmitter levels^19^ and/or fish behavior. Exposure to oxazepam, for example, has been causatively linked to behavioral alterations in some fish species.^37−39^ Various antidepressants may affect fish behavior as well. Examples include disruptions in anxiety- and aggression-related behaviors in zebrafish (*Danio rerio*) and Siamese fighting fish (*Betta splendens*) exposed to fluoxetine^40,41^ and suppressed foraging behavior in zebrafish exposed to escitalopram.^42^

Although evidence exists that some human pharmaceuticals can affect the physiological functioning and/or behavior of individual fish, very little is known about their impact at population level. Indirect impacts of APIs on fish via their effects on other trophic groups, and vice versa, have been indicated,^28^ but this is not the focus of this review.

## Susceptibility of Fish to Human APIs in the Environment – Extrinsic Factors

### Pharmaceutical Fate in Aquatic Environments

The environmental fate and behavior of pharmaceuticals in aquatic systems will vary considerably, both spatially and temporally, as a result of wide-ranging environmental variables, including water quantity, temperature and physico-chemistry.^13,43^ Pharmaceuticals may remain unchanged, undergo biotic and/or abiotic transformation, sorb/bind to suspended matter, dissolve in water and/or accumulate in biological tissues,^6,44^ all of which may affect their bioavailability and potency in exposed fish. Some drugs can travel long distances from their source(s),^5^ particularly when they have long degradation half-lives and low organic carbon/water coefficients (log *K*_OC_ < 4.0 at pH 4–9), enhancing their environmental persistence and mobility.^45^ The continuous release of pharmaceuticals into waterways can moreover cause the exposure profiles of degradable drugs to mimic those of truly persistent pollutants–a phenomenon known as pseudopersistence.^3,7^

While many APIs survive biodegradation and enter receiving waters as parent compounds, others are generated as metabolites and reactive intermediates. Some of these entities may be equally or even more potent than their parent compounds, as is the case for salicylic acid and norfluoxetine, the active metabolites of aspirin and fluoxetine, respectively.^46^ Different chemical species of the same compound, such as diclofenac and its nitroso derivative, may also elicit synergistic toxic effects.^47^ Furthermore, conjugated biotransformation products of some APIs may be deconjugated by microbial enzymes in WWTPs, rendering them biologically active again.^48^

APIs and their metabolites can participate in various chemical and biochemical reactions in the environment that may affect their bioavailability and/or biological potency. At least half of all pharmaceuticals in current use may undergo chiral inversion, for example, causing one enantiomer to predominate in terms of environmental occurrence and toxicity.^49−52^ Additionally, pharmaceuticals in surface waters may be subject to phototransformation, resulting in products with either lower or higher toxicity potential, the latter of which has often been noted for NSAIDs such as diclofenac.^53^ While warmer water temperatures can accelerate API biodegradation, it may, conversely, amplify the bioactivation and toxicity of some pollutants by altering homeostatic processes.^54^ The interaction of pharmaceuticals with dissolved organic matter has also been shown to affect bioavailability, either enhancing^55^ or reducing^56^ drug accumulation and toxicity in exposed aquatic organisms.

### Physico-Chemical Properties of APIs Affecting Their Bioavailability in Fish

**Figure 1:**
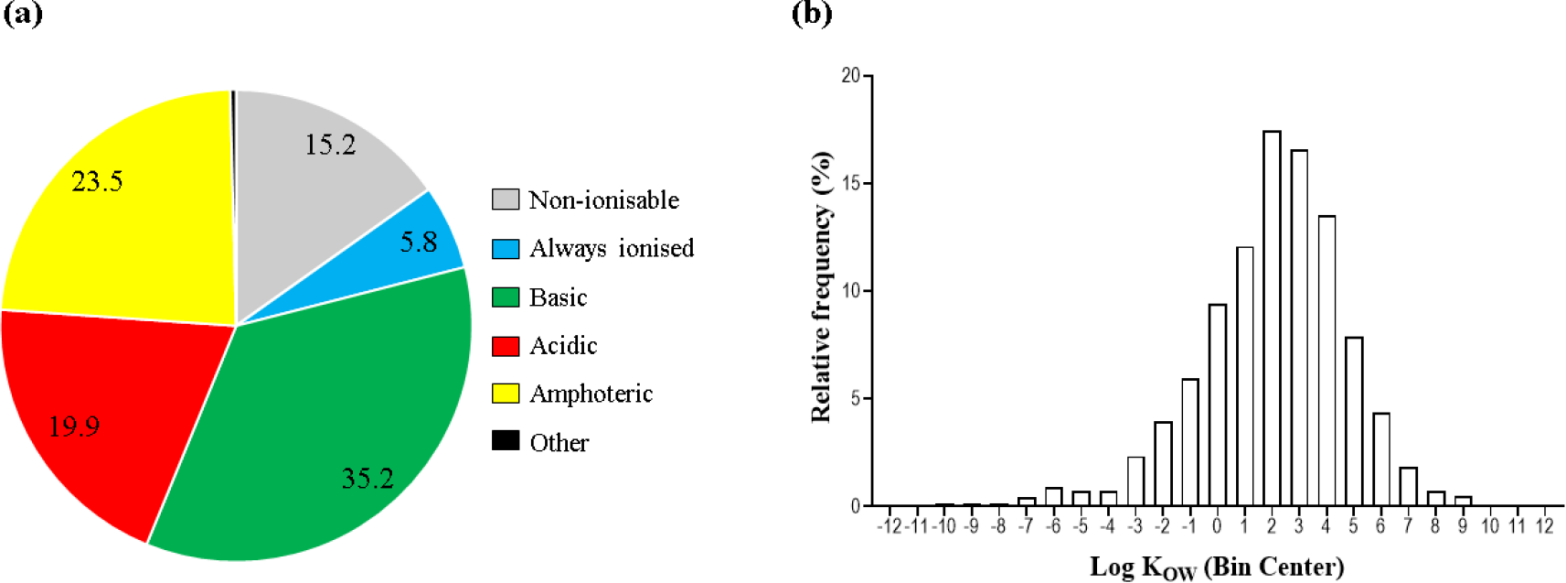
Overview of the chemical space occupied by pharmaceuticals based on their (a) ionization properties (according to Manallack^66^) and (b) hydrophobicity coefficients (according to Berninger and colleagues^24^). Abbreviations: log *K*_OW_, *n*-octanol/water partition coefficient.

Currently approved pharmaceuticals occupy a very broad “chemical space” in terms of their molecular and associated physicochemical properties (Figure 1) which, as key aspects of drug ADME, will result in different bioaccumulation and toxicity potentials. The potential for an API, as for all chemicals, to bioaccumulate in fish is generally characterized by its bioconcentration factor (BCF), where *C*_fish_ and *C*_water_ are the chemical concentrations in the organism and water at steady state, respectively:
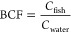
1

ERA requires BCFs for APIs with bioaccumulation potential (i.e., an *n*-octanol/water partition coefficient, log *K*_OW_, ≥3),^57,58^ a criterion which is breached by about 54% of currently approved pharmaceuticals (Figure 1b), many of which are yet to be tested. Consequently, although the data currently available suggest that most of the tested pharmaceuticals pose a low bioaccumulation risk to aquatic organisms, empirical BCF data are lacking, notably for anticancer drugs and API metabolites more generally.^59^ The tests required to determine BCFs are time-consuming, costly and require large numbers of fish to be sacrificed (100–200 individuals or more for a single full aqueous exposure bioconcentration test).^60^ Moreover, experimental BCF data on the same drug are often variable between and within studies, particularly for highly lipophilic compounds.^61^ Researchers have consequently started looking into machine learning methods to predict BCF values based on key physicochemical drug properties. Molecular weight (MW) and lipophilicity (represented by log *K*_OW_ for neutral compounds), for instance, are both inversely related to water solubility, which affects the amount of drug freely available for absorption.^62^ With increasing log *K*_OW_ values (up to a value of 5), there is also increased partitioning into lipophilic biological membranes and tissues, thereby facilitating uptake and accumulation.^63^ For ionizable compounds, correcting the *K*_OW_ by the fraction of neutral molecules or using the pH-dependent distribution coefficient (log *D*) as an input parameter have been suggested as possible ways to improve BCF predictions.^64^ Considering the assumptions implied in this approach as well as the inherent differences between natural fish lipids and octanol, however, the membrane/water (log *K*_MW_) or liposome/water partition coefficient (log *K*_LipW_) may be more reliable surrogates in this regard.^61,65^ Nevertheless, log *D* takes into account ionization state, which is dependent on the acid dissociation constant (p*K*_a_) – another important determinant of drug fate and bioavailability, especially considering that the majority of pharmaceuticals contain an ionizable group (Figure 1a).^66^ While both neutral and ionized species are believed to contribute to the passive uptake of APIs through the establishment of a concentration gradient, ions are less likely to cross lipid membranes and hence the effect of environmental pH on ionization is critically important.^56,67^ Likewise, the pH of body fluids will determine the degree of electrolyte dissociation within an organism, which will ultimately affect how these compounds, particularly those with p*K*_a_ values of approximately 5–9,^68^ are dealt with by the body and interact with drug targets. In addition to MW, log *D* and p*K*_a_, topographical polar surface area and the number of nitrogen atoms have also been noted as important molecular descriptors to keep in mind when making BCF predictions.^69^

In support of the aforementioned, Chang and colleagues^70^ applied a partial-least-squares regression model to predict pharmaceutical uptake rate across an *in vitro* fish gill system and found that log *D*, MW and p*K*_a_ were some of the most significant drivers. This provides substantial evidence that models based on a combination of physicochemical drug properties can be useful in understanding pharmaceutical uptake and accumulation in biota and, in conjunction with other *in vitro* and *in silico* tools, could potentially replace or at least significantly reduce the number of whole animals used in bioaccumulation studies.

## Susceptibility of Fish to Human APIs in the Environment – Intrinsic Factors

In addition to environmental factors and drug-related properties, certain intrinsic physiological factors underlying drug absorption, distribution, metabolism, excretion and toxicity (ADMET) may render some fish more or less susceptible to the exposure and effects of pharmaceuticals than other fish or mammalian species. Here, we identify these factors to help highlight where fish testing is likely to be required in ERA to ensure the optimal protection of fish populations. Particular attention is given to teleost fish, which represent the majority (>26,000) of extant fish species (>30,000) and more than half of all extant vertebrate species.^71^

When assessing the risks of pharmaceuticals to nontarget organisms, one should first and foremost discern the potential for target interaction and resultant pharmacological effects in the organism of interest. We hence start the ADMET intrinsic analyses for API effects in fish with T (toxicity) and then consider whether the ADME properties of relevant drugs are likely to increase or decrease the risk of adverse effects.

### Toxicity (T) Related to Drug Target Conservation and Off-Target Interaction

Traditionally, pharmaceuticals have been designed to modify physiological function by interacting with a particular target via a specific mode of action (MOA). As these targets are often highly conserved across vertebrate animal phyla,^12,72,73^ drugs designed to induce therapeutic effects in humans may be biologically active in certain species of wildlife. Studies have shown that between 65 and 86% of human drug targets are evolutionary conserved across a number of fish species.^72−74^ Such well-conserved targets are associated with a higher likelihood of drug-target interaction, pharmacological effects and, potentially, toxicity, assuming that the resultant pharmacological effects in nontarget organisms (such as fish) occur at lower concentrations than toxic off-target (adverse) effects.^75^ Even so, drugs are not 100% target-specific and may interact with off-targets and/or homologues in fish that, as a result of the process of genome duplication,^76^ may differ from those in humans.^3,6,77^ Binding of pharmaceuticals to these sites may lead to unintended (and unexpected) physiological effects with potentially detrimental outcomes.

Adding to the complexity, multitarget drugs have recently attracted attention as promising tools to fight challenging diseases such as malaria, cancer, tuberculosis and diabetes.^78^ As these APIs act via multiple MOAs, they may pose different and/or additional risks to nontarget species. Another class of high-risk pharmaceuticals is those with conserved MOAs or additive effects, which include the corticosteroids.^79^ Although the environmental concentrations of individual drugs in such classes might not be sufficient to induce effects on their own, environmentally relevant mixtures may conjointly elicit pharmacological effects in fish.^22,80,81^

The potential effects of APIs designed to target the immune system are a further area of concern. Unlike mammals, fish heavily rely on their innate (nonspecific) immune system for survival during the early stages of embryogenesis.^82^ In later life stages, this system remains crucial in supporting adaptive immune responses, which are limited by fish’s cold-blooded nature, limited range of antibodies and slow lymphocyte proliferation.^83^ An increased hepatic gene expression of C-reactive protein (c7), which forms part of the complement system and participates in both innate and adaptive immunity,^84^ appears to be a common effect of NSAID exposure in fish, as shown by a clear concentration-dependent response to both naproxen and diclofenac.^85−87^ By altering such important components of the immune system, immunomodulatory drugs may increase the susceptibility of fish to infections and, potentially, the toxic effects of other drugs.

While drug toxicity testing (via various animal-based and alternative methods) is still ongoing, data sets (e.g., by Gunnarsson et al.^88^) and databases (e.g., the ECOTOX Knowledgebase^89^) have been established to capture and maintain up-to-date ecotoxicity data for chemicals, including pharmaceuticals, in aquatic organisms.

### Physiological Processes Affecting ADME of APIs in Fish

Most research on the potential effects of human pharmaceuticals in fish has focused on drug-target interaction. Knowledge on how fish actually process different drugs and how their distinctive physiological features and functions affect their ability to do so has received less attention, yet will have fundamental bearing on the likelihood for any adverse effects. Understanding how much of the drug reaches the site(s) of action (i.e., bioavailability), how and when this will occur (i.e., PK), and to what extent the drug and its metabolites will accumulate in the body (i.e., bioconcentration and bioaccumulation) are key factors underlying the susceptibility of fish to certain groups of pharmaceuticals and are governed by the processes of absorption, distribution, metabolism and excretion (ADME).

### Absorption (A)

Uptake or movement of a drug from the environment into the systemic circulation may occur via various routes in fish, including the skin (dermal), gut (dietary) and gills (branchial). The oral route is the main route of administration for most human drugs, requiring solubilization, permeation and absorption of the drug via the gastric mucosa into the bloodstream. As a result, the vast majority of orally active drugs conform to the “rule of five”, i.e., they have a MW ≤ 500, calculated log *K*_OW_ ≤ 5, H-bond donors ≤ 5 and H-bond acceptors (sum of N and O atoms) ≤ 10.^90,91^ These properties may also facilitate drug absorption across epithelial membranes in fish more generally. API uptake in fish is most likely to occur for pharmaceuticals that are sufficiently water-soluble to remain in the aqueous system but also lipophilic enough to diffuse across lipid membranes. However, fish are capable of absorbing both hydrophilic and hydrophobic compounds. Hydrophilic APIs tend to persist in aqueous environments and are taken up by fish mainly from the water across the gills or skin surfaces (bioconcentration). Hydrophobic APIs are most likely to be taken up via a dietary route (bioaccumulation) or even maternally from the parent to the developing embryos.^92,93^ Alternatively, hydrophobic drugs may be taken up directly from the water column when they adsorb to particulate organic matter and the resulting complexes interact with the gill mucosa.

Regardless of the uptake route, the absorption of pharmaceuticals in fish involves transport across multiple biological membranes – dynamic structures consisting of lipid bilayers interspersed with lipid rafts, oligosaccharides, proteins and glycoprotein complexes,^94^ the composition and structure of which have a major bearing on their permeability to APIs.^61,95^ In fish, inter- and intraspecies differences in membrane fatty acid composition may not only be genetic but may also be affected by short and long-term adaptations to environmental conditions such as water temperature, salinity and/or pH.^96^

For most APIs, uptake in fish predominantly occurs via a multistep process across the gill,^75,97,98^ which is facilitated by this structure’s large surface area, rich blood supply, short diffusion distance between water and blood, and a wide array of transport proteins and ion channels that facilitate the passive and active transport of chemicals.^68^ The branchial uptake of pharmaceuticals is affected by fish’s ventilation and heart rates,^99,100^ with higher activity levels and drops in water pH (to around pH 4)^101^ potentially increasing the risk of absorption. Water salinity has also been shown to influence pharmaceutical uptake across the gills, most likely as a result of its effect on the oxygen consumption rate.^102^ Additionally, seasonal and diurnal fluctuations in dissolved oxygen and carbon dioxide (CO_2_) levels, which can be particularly pronounced in freshwater (FW) systems, can trigger physiological responses in fish that can potentially affect pharmaceutical uptake and tissue bioavailability.^103,104^

Fish are capable of modifying the chemistry of the water they breathe by extracting oxygen and ions, while releasing CO_2_, ammonia (NH_3_) and metabolic products at the gill surface. The resultant pH adjustments serve a protective role by buffering the gill microenvironment, but are also important determinants of the fate of ionizable compounds, including many APIs. Accordingly, Chang, Town and colleagues^105^ noted that fluctuations in water pH (between pH 6–8) could lead to large variations in uptake of both the weak acidic API ibuprofen and the weak base propranolol. Propranolol (p*K*_a_ 9.45), as an example, is predicted to be 99.9% ionized at pH 6, but only 96.6% ionized at pH 8 (eq 2), hence allowing it to be more easily absorbed at the latter (higher) pH. Still, ions play an important role in passive branchial uptake by participating in diffusive flux and maintaining steep diffusion gradients for neutral molecules across membranes.^106^ Other important determinants affecting passive API uptake across the gill are aqueous exposure concentration, compound MW (which generally shows a negative correlation with uptake rate)^70^ and lipophilicity, where chemicals with either relatively high (>6) or low (<3) log *K*_OW_ values tend to be less bioavailable via uptake across the gills, mainly as a result of partitioning to nonaqueous systems (i.e., sewage sludge, soil and sediment) or low diffusion gradients (driven by weak interactions with blood components), respectively.^68,107^ Trans-epithelial potential, which can be influenced by factors such as water pH and calcium content,^108^ can also affect the rate of branchial API uptake. Taken together, fish will likely be most susceptible to the branchial uptake of drugs with low MW and moderate lipophilicity, while the absorption of ionizable compounds will additionally be influenced by drug p*K*_a_ as well as the pH of water and body fluids.
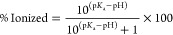
2

**Figure 2:**
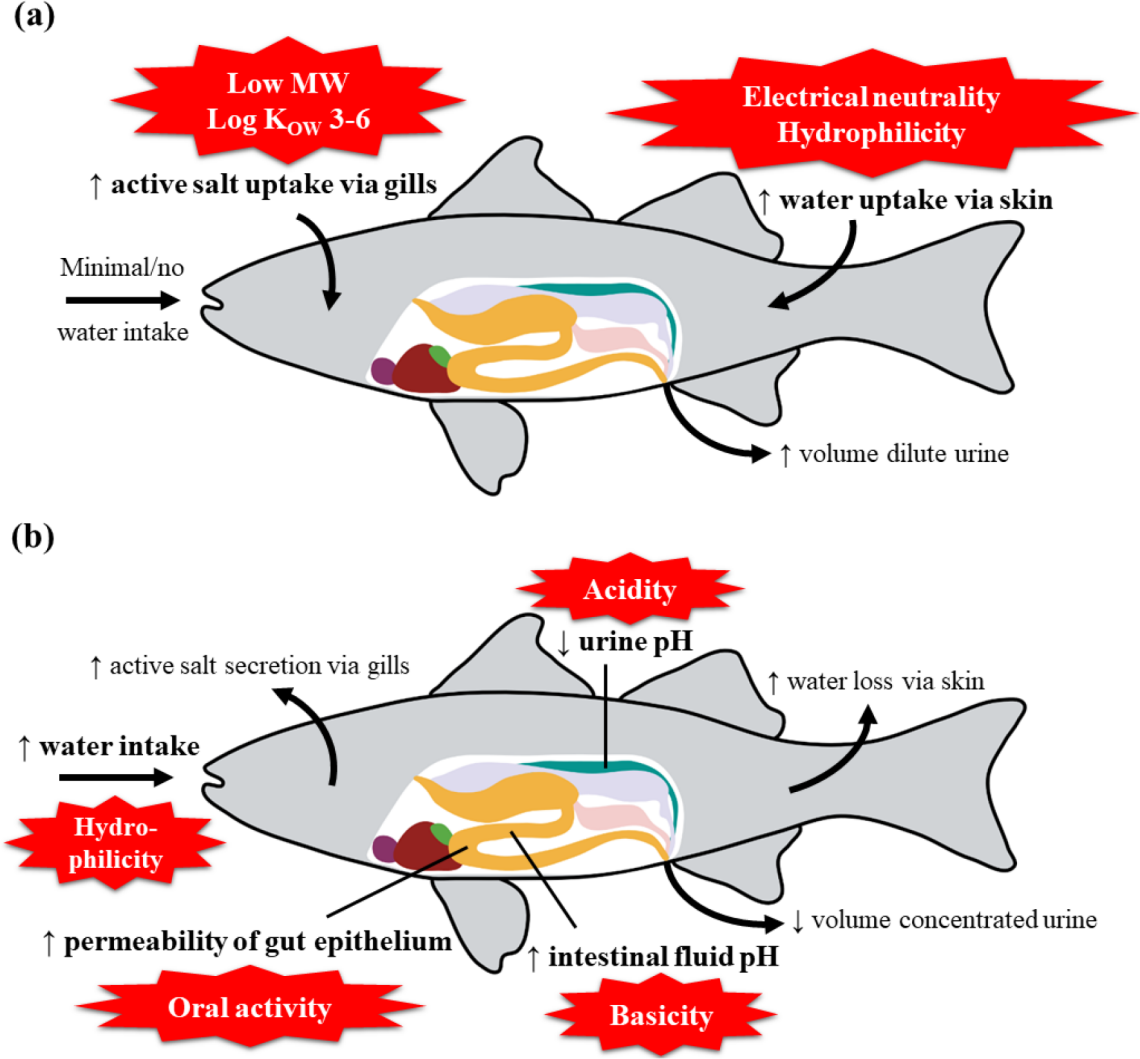
Physiological features of (a) freshwater and (b) saltwater fish that may increase susceptibility to pharmaceutical exposure and effects (indicated in bold). Drug properties associated with increased risk of uptake are indicated with red stars. Abbreviations: log *K*_OW_, *n*-octanol/water partition coefficient; MW, molecular weight.

In addition to passive diffusion, active concentration-independent transport, mediated by carrier proteins, can also contribute to the uptake and disposition (see Distribution) of pharmaceuticals in fish. It has been shown, for example, that propranolol is absorbed across the rainbow trout gill tissue via a combination of passive and carrier-mediated mechanisms.^105,109^ The active uptake of pharmaceuticals via the gills may be particularly important in FW species; these fish are hyper-osmotic to their environment and therefore need to use their gills to absorb salts against a concentration gradient (Figure 2a).

ATP-binding cassette (ABC) transporters play a significant role in chemical uptake and elimination in fish. The presence of all primary vertebrate ABC drug transporters (or their homologues) has been confirmed in fish tissue, including those associated with drug uptake and clearance, such as the gills.^110−112^ Many nuclear receptors involved in the transcriptional regulation of these transporters have also been identified in fish. The pregnane X receptor (PXR), for instance, is expressed in the gut and gill tissues of Japanese pufferfish (*Fugu rubripes*).^113^ While fish ABC gene sequences resemble those in mammals, suggesting similar distribution patterns, functional properties and physiological roles in both vertebrate groups,^114^ transporter expression and substrate specificities may vary between species,^115^ complicating the prediction (via biological read-across) of drug partitioning and uptake in fish from mammalian data. For example, chemical uptake across the rainbow trout gill appears to occur in a more passive manner compared to that in the mammalian lung, with the gills having been characterized by low basal expression levels of ABC transporter genes.^116^ Transporter expression may additionally be subject to change over the course of the fish’s life cycle,^30^ resulting in intraspecies variability. Drugs known to be transporter substrates in humans might therefore pose differential risks to different fish species and life stages given that variations in transporter expression and specificity may alter drug absorption (also see Distribution and Excretion).

Dietary uptake of pharmaceuticals has been shown to be less important than branchial uptake in wild fish.^97^ Nevertheless, the fish gastrointestinal tract (GIT) shares significant structural similarities with that of mammals^68,117^ and thus the design features of human APIs intended for oral administration will likely operate similarly in fish. Making any generalizations for fish, however, is difficult as the digestive system can vary substantially between different species with different feeding strategies^100^ and even between different life stages of the same species. Furthermore, factors such as water temperature affect feeding and digestion rates,^100^ and directly impact conditions within the GIT, such as pH^118^ and intestinal microbiota composition,^119^ all of which may affect the dietary uptake of drugs.

Marine teleost fish have a number of physiological features that may render them more susceptible to the uptake of APIs from the GIT than FW fish (Figure 2b). First of all, being hypo-osmotic to their environment, they consume large volumes of seawater for osmoregulatory purposes.^120,121^ Second, cortisol stimulates cellular apoptosis in the GIT of fish acclimating to saltwater (SW), thereby making the epithelium more permeable than in FW fish.^122^ Substantial amounts of bicarbonate ions are also secreted into the intestines of marine fish, precipitating divalent cations such as calcium and thereby further promoting the absorption of water.^123^ As a result, the intestinal fluids of these fish (at pH 8.4–9)^124^ are far more alkaline than that of mammals and their FW counterparts (pH 6–8).^125,126^ In theory, this phenomenon widens the pH range to which pharmaceuticals may be exposed along the entire length of the gut and, in turn, may facilitate the uptake of basic compounds from the intestines of SW fish. Equally, however, marine environments will generally have lower exposure levels as a result of dilution, limited drug transport from estuaries and harbors to the open sea,^127^ and the “salting out” effect of SW on certain pharmaceuticals.^128^

Despite the fact that the skin is in constant contact with water, dermal uptake of pharmaceuticals in fish is arguably overlooked during risk assessments. This route usually contributes to <10% of total uptake in large fish^129^ but may potentially account for up to half of the total drug uptake in some fish species and/or life stages. In humans, transdermal drug delivery requires drugs to have a low MW (MW < 500), a balanced lipophilicity (log *K*_OW_ 1–3), and some solubility in both oil and water.^130^ Fish lack the keratinized epidermal layers seen in mammals,^131^ making the diffusion pathway across their skin mainly aqueous^132^ and, hence, potentially accessible to a broader range of APIs. To illustrate, the topically active antifungal terbinafine is unable to penetrate human skin due to its high affinity for keratin,^133^ but has shown the ability to cross the skin in zebrafish.^134^ Konrádsdóttir et al.^135^ also demonstrated that both hydrophilic and lipophilic molecules (log *K*_OW_ < −3 to 5.1) can permeate catfish skin via a diffusion-controlled process. In fish, the dermal route may be particularly important for the uptake of neutral molecules in embryo-larval stages, juveniles (before the gills are fully functional) and in some small species with large cutaneous surface area-to-volume ratios and thin, highly vascularized skin,^136,137^ as well as in scaleless species, such as the channel catfish (*Ictalurus punctatus*).^129^ Additionally, demersal/benthic fish species may be particularly vulnerable to the uptake of sediment-associated compounds across the skin.^132^ In theory, FW fish may generally be more susceptible to the dermal uptake of certain drugs than SW species due to the higher levels of water being absorbed through their skin (Figure 2a), but this will also depend on other factors, including the chemical nature of the drug.

### Distribution (D)

Following absorption, drugs distribute into interstitial and intracellular fluids to different extents. This process is largely dependent on the relative affinity of the particular drug for the blood and different body tissues. Whereas the mammalian circulatory system is divided into three circuits (pulmonary, coronary and systemic), fish possess a single blood flow circuit whereby blood from the gill is directly pumped to the rest of the body before returning to the heart.^138^ Blood entering the body tissues is consequently at a lower pressure compared to that in mammals, but how this might affect drug distribution is not clear.

Only a handful of studies have investigated the distribution of pharmaceuticals within the bodies of exposed wild fish^139,140^ where drug accumulation is compound- and species-specific.^5,141^ Instead, the majority of distribution data available for pharmaceuticals in fish are from time-course laboratory bioconcentration studies. In one such study, atenolol and venlafaxine displayed tissue-specific distribution in zebrafish, with bioaccumulation directly correlating with the lipid content of each tissue.^142^ Predictably, accumulation potential was shown to be governed by drug hydrophobicity, with venlafaxine (log *K*_OW_ 3.28) accumulating to a greater extent than atenolol (log *K*_OW_ 0.16) in the studied tissues. Importantly, the molecules of both these APIs are small enough to cross the blood–brain barrier (MW < 400) and accumulate in the lipid-rich brain tissue as well.^142,143^ Hydrophilic APIs may also exhibit tissue-specific toxicity, as seen for the nephrotoxic aminoglycosides (log *K*_OW_ < −3), which accumulate in the kidneys.^144^ Like absorption (see Absorption), drug partitioning is influenced by transmembrane pH and electrical gradients, as well as membrane composition and active efflux processes.^61,112^

The binding of drugs to plasma components–mainly proteins, but also lipids and glycoproteins–is another major factor influencing the PK process of distribution.^145^ The total bound fraction is primarily a function of the drug concentration, the number of protein and lipid binding sites present and their affinity for each other.^146^ Drug–protein complexes are generally too large and polar to cross cell membranes, hence constituting inactive reservoirs that either prolong drug action by circumventing metabolism and/or excretion, or limit drugs’ ability to reach their target sites. The total plasma protein content in both rainbow trout^147^ and zebrafish^148^ seem to be lower than that of humans. Furthermore, it has been shown that acidic pharmaceuticals, such as the NSAIDs naproxen and ibuprofen, tend to bind less strongly to fish than to human plasma (>70× less for naproxen), making them more bioavailable in fish, whereas weak bases such as propranolol seem to bind to a similar extent in both plasma types.^147,149^ This varied binding can likely be attributed to a lack of high-affinity binding sites for organic acids on fish plasma proteins, which may partly be a consequence of the difference in blood pH between humans (pH 7.35–7.45)^150^ and fish (pH 7.3–8).^151^ Alternatively, it may result from interactions or competition between drugs and endogenous ligands, such as fatty acids, for protein binding sites.^152^

The major plasma protein in humans, human serum albumin (HSA), serves as a carrier for numerous endogenous and exogenous molecules, including most acidic and neutral drugs.^146^ The structure of HSA is highly adaptable and may undergo conformational transitions in response to changes in blood pH and chemical exposure, consequently affecting ligand affinity.^146,153^ In fish, albumins are present in the plasma of several species at levels ranging from <10% up to almost 60% of total plasma protein^154^ and show huge structural, and hence functional, diversity.^155^ Plasma albumin in rainbow trout, for example, has been described as “para-albumin” due to its significant functional differences from HSA.^156^ In some fish species, including the cyprinids zebrafish^157^ and carp,^158^ albumin-like plasma proteins seem to be completely absent. Indeed, the exact role of this carrier protein in fish plasma is still unknown. α_1_-Acid glycoprotein (AGP) is another prominent protein that exists in up to 20 different forms in human blood plasma where it is involved in the binding and transport of various basic and neutral lipophilic drugs, as well as some acidic drugs.^159^ As for HSA, blood pH and chemical exposure have an important impact on the degree of drug binding to AGP.^160^ Fish species commonly used for ERA, including zebrafish, carp and rainbow trout, also seem to lack AGP,^149^ but there is limited research on this more widely in fish.

In addition to HSA and AGP, various apolipoproteins (proteins that bind to lipids) also contribute to the plasma binding of pharmaceuticals. Indeed, these molecules have been shown to act as alternative carriers in fish when plasma protein levels are low.^158^ Compared to humans, most Teleostei are considered hyper-apolipoproteinaemic. For example, while apolipoproteins make up 36% of all plasma proteins in rainbow trout, this fraction is about three times lower in humans.^161,162^ It is therefore likely that apolipoproteins have a greater impact on the bound fraction of pharmaceuticals in fish than in humans, which will ultimately affect drug binding kinetics and bioavailability. In support of this, there seems to be a good correlation between percentage plasma protein binding (PPB) in fish and log *K*_OW_ values, particularly for anionic compounds.^149^ Interestingly, significantly higher levels of plasma lipids have been observed in temperate-water fish than in Antarctic species,^163^ hence suggesting that the contribution of apolipoproteins and lipids to drug binding may further vary between fish species as a result of environmental adaptation. Nevertheless, considering that so much is still unknown regarding PPB in fish and the fact that it seems to be so variable among different species, direct read-across based on the assumption that drug PPB is equivalent in fish and mammals can only be applied when exercising great caution and accepting major uncertainties.^149,164^ As small differences in binding may result in substantial variations in effects and/or toxicity, drugs displaying high degrees of PPB in humans, especially weakly acidic and/or low *K*_OW_ compounds, should be prioritized during risk assessment. This may particularly be important for narrow therapeutic index drugs, which already tend to display high intersubject variability in PK and PD parameters.

The fish plasma model (FPM) is being increasingly applied as a read-across approach, comparing a measured human therapeutic plasma concentration (H_T_PC) to a predicted fish steady-state plasma concentration (F_SS_PC) to compute an effect ratio (ER = H_T_PC/F_SS_PC) that indicates the potential for human drug target-mediated effects in fish.^165^ According to the FPM, the closer a drug’s F_SS_PC is to H_T_PC, the greater the potential for a pharmacological effect in fish.^164^ Consequently, an ER value <1 would be indicative of a potential risk. The FPM, however, makes a number of inherent assumptions^147^ that may not necessarily hold true due to differences between fish and mammals in terms of blood pH and the involvement of plasma components in drug PK (as described above), giving a cautionary note to its application. Still, it remains a useful tool for supporting the screening of priority pharmaceutical pollutants and, with refinements and additional data, has the potential to be a very useful tool in the risk assessment for APIs in fish (see Table S1, Supporting Information 1).

Body lipid content and the dynamics of lipid turnover may have significant effects on the distribution, tissue storage and metabolism of lipophilic drugs in fish, with the likelihood of seasonal spikes in drug plasma levels as lipid reserves are mobilized during colder months and periods of high energy usage.^166^ As an example, liver triglyceride stores have been shown to be 54% higher in summer- than winter-acclimated yellowbelly rockcod (*Notothenia neglecta*).^167^ It is also worth noting that APIs released from female fish’s fat stores during spawning may be voided into their eggs,^92^ presenting a risk to sensitive embryonic and larval developmental stages.

ABC transporters (also see Absorption and Excretion) play an important role in determining the entry and expulsion of molecules to and from different body compartments and thus affect drug distribution. Most studies on these carrier proteins focus on ABCB1, otherwise known as P-glycoprotein (P-gp). P-gp is remarkably catholic with regards to its substrates and is believed to act as a first line of defense (phase 0 detoxification) against unmodified compounds in both mammals and fish. P-gp’s efflux activity may influence the effective pharmacological dose of drugs, as the dose has to exceed the transporters’ capacity before sufficient drug can enter the cell and elicit a therapeutic (or toxic) effect.^168^ Many studies have assessed P-gp’s distribution patterns in fish tissues,^110^ but data regarding the distribution of other ABC efflux transporters are limited due to the lack of appropriate fish-functional antibodies. P-gp’s lack of specificity may not always be beneficial for nontarget organisms. While some compounds can induce the expression of efflux transporters, many chemicals can also inhibit their activity, thereby enhancing toxicity, a phenomenon known as chemo-sensitization.^168^ A large number of chemical substrates have been shown to promote similar ABCB1-ATPase *in vitro* activity in fish and human liver cells, but differences have also been reported,^169^ such that drugs that are subject to efflux in mammals (i.e., P-gp substrates) will not necessarily be recognized by efflux transporters in fish and may thus be more likely to accumulate.^168^

### Metabolism (M)

Drug metabolism generally involves processes that transform lipophilic chemicals into more hydrophilic entities, thereby facilitating their elimination from the body via urine or bile (see Excretion). In fish, as in mammals, this process is catalyzed by different groups of enzymes across two phases.^145,170−173^ Both phase I and II enzymes are widely distributed throughout the fish body and may subject significant fractions of APIs to metabolism within the gill, gut, liver and kidney, thereby reducing compound bioavailability and affecting the level of environmental exposure required to elicit pharmacological and toxic effects.^173,174^

*In vivo* studies on pharmaceutical metabolism in fish are limited but indicate that they are capable of metabolizing drugs in a similar manner to humans (see Table S2, Supporting Information 1). For some APIs, however, there appear to be differences in terms of metabolites formed and, thus, associated metabolic pathways.^175−177^ Drawing parallels between drug metabolism in fish and humans is further complicated by apparent differences in results obtained from different fish species and/or experimental approaches.^176,178−180^

Cytochrome P450 enzymes (CYPs), particularly those belonging to families 1 to 3, are the most significant enzymes involved in pharmaceutical metabolism in humans,^181^ where their activities are largely controlled by ligand-activated receptors, such as the aryl hydrocarbon receptor and the PXR. Numerous drug-metabolizing CYP isoenzymes (the majority of which seem to belong to the CYP1 and CYP3 families) and their respective nuclear receptors have also been identified in fish,^173^ but knowledge concerning the exact role of each enzyme family in these organisms is still limited. Genetic and environmental factors may moreover give rise to wide variations in drug metabolic pathways and overall metabolic efficiency between different fish species.^182^

**Figure 3:**
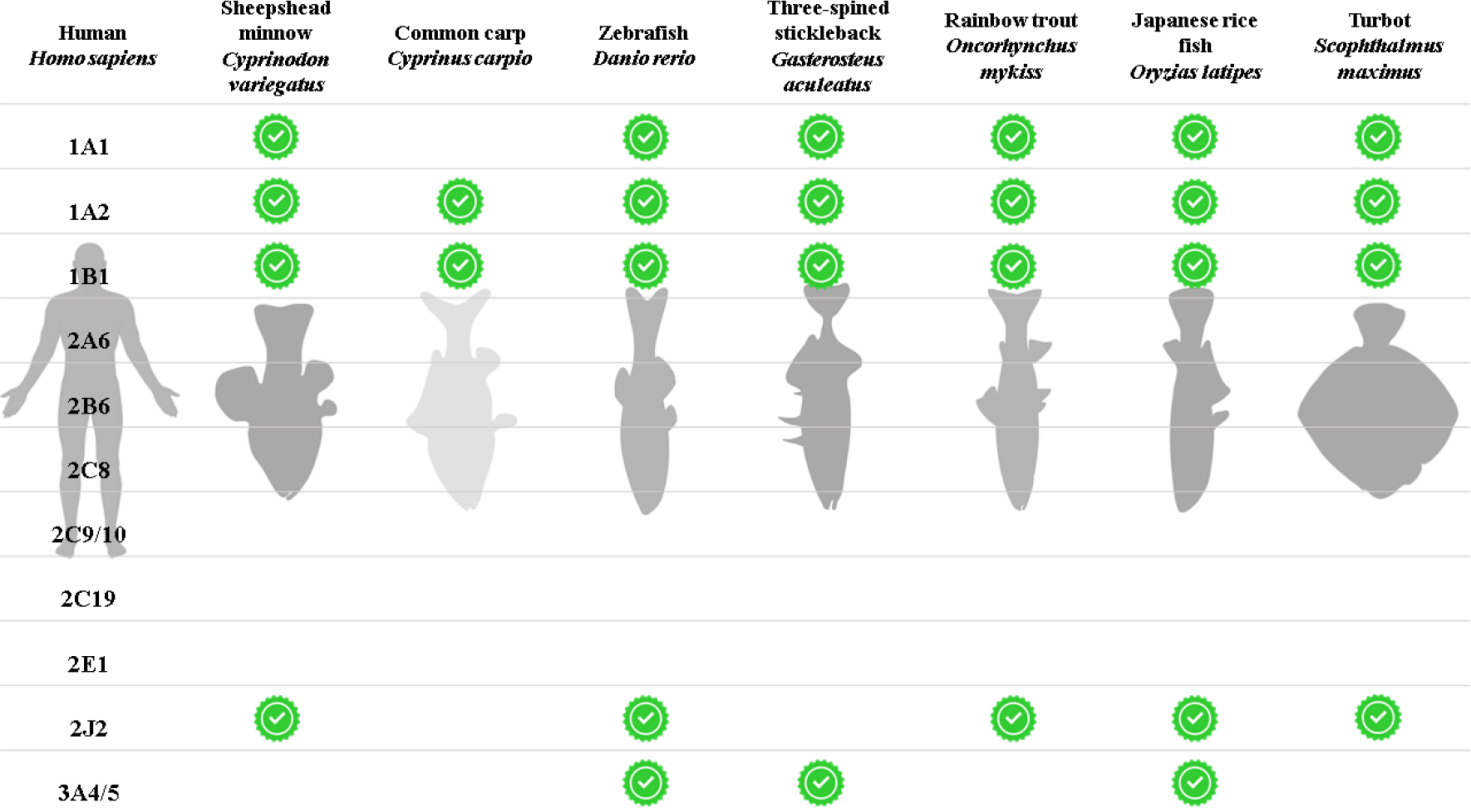
Presence of predicted orthologs for major human cytochrome P450 genes involved in drug biotransformation in fish species with sequenced, well annotated genomes. Green ticks indicate the likely presence of an ortholog through majority scores across four online ortholog prediction tools (EggNOG, Ensembl, Inparanoid and ORCAN).^194−197^ Predictions for *Cyprinus carpio* are based on results from Ensembl only. See Supporting Information 2 for detailed results.

Some drugs can interfere with pharmaceutical metabolism by either altering the expression of metabolic enzyme-associated genes or by directly binding to and promoting or inhibiting enzyme activity. The anticonvulsant drug carbamazepine, for example, is a strong inducer of CYP2B6 and CYP3A in human patients,^183^ thereby enhancing the metabolism of these enzymes’ substrates (including itself). Considering that nontarget organisms, including fish, are generally exposed to pharmaceuticals as multicomponent mixtures, such interference can ultimately result in drug–drug interactions and in turn unforeseen adverse effects, including impaired homeostasis and even toxicity. The induction and inhibition of fish CYPs are generally believed to follow similar mechanisms to that seen in mammals, but significant variations in enzyme (or ortholog) expression (Figure 3), activity and substrate specificity may still be apparent.^184−189^ As an example, clotrimazole, a potent CYP3A4 inhibitor/inducer in humans, was not found to affect the expression of any relevant genes in a carp primary hepatocyte model.^190^ In another study, ketoconazole was confirmed to be a potent inhibitor of both CYP1A and CYP3A activity in rainbow trout, while it selectively inhibited CYP3A in killifish.^184^ Furthermore, pharmaceuticals known to be CYP inhibitors in mammals were indeed shown to inhibit CYPs in zebrafish and rainbow trout, albeit with a much broader target selectivity.^191,192^ Some species of the Loricariidae family (armored catfishes) have also been seen to display some perplexing CYP1A substrate selectivity.^193^ The interaction between CYP substrates, inducers and inhibitors and their receptors in fish therefore seems to be highly complex, as well as both tissue- and species-specific,^173^ making prediction or read-across from mammalian data difficult. In addition, although fish might still be able to metabolize certain drugs for which they lack the relevant human CYPs or orthologs (notably from the human CYP2 family), assuming this for all human CYP substrates is unfounded and likely to lead to underestimated toxicities in fish. Taken together, there is a pressing need for further research on the conservation, tissue-specific expression, activity and specificity of drug-metabolizing CYPs in different fish species.

While metabolism is widely assumed to lead to detoxification, metabolic enzymes are also capable of activating drugs and/or increasing their toxicity. Prodrugs, for instance, remain essentially inert until they are transformed to their active metabolites. On the other hand, the metabolism of some already active pharmaceuticals can lead to the formation of reactive intermediates that may disrupt cellular function or initiate untoward immune responses.^198^ The presence of reactive and potentially harmful substructures in molecules, such as catechol groups, quinones and fluorine,^199,200^ may help account for such “enhanced toxicity” by making compounds more likely to participate in chemical and biochemical interactions. These reactions may have direct toxic effects (i.e., carcinogenicity or mutagenicity) or lead to the formation of toxic byproducts, including reactive oxygen species.^201,202^ The fine balance between these two alternatives (detoxication vs bioactivation) is a key determinant of inter- and intraspecies differences in toxicity and will most likely lead to the production of different concentrations of (re)active metabolites in mammals and fish. To illustrate, whereas the metabolism of the benzodiazepine temazepam in humans produces a negligible amount of oxazepam, significant accumulation of this active metabolite has been observed in fish tissues following the exposure of European perch (*Perca fluviatilis*) to the parent compound.^203^ Consequently, drugs known to have promiscuous functional groups and/or (re)active metabolites should be prioritized during both human safety and environmental risk assessments, and caution exercised when attempting to extrapolate data from mammalian studies for such compounds.

The metabolites of some pharmaceuticals may have ecological significance, but many of these are yet to be identified^46^ and thus have not been risk assessed. Information on bioactivation pathways in mammals can serve as a guide on the metabolites most likely to be introduced into the environment and existing toxicity data may then further aid in determining which of these should be targeted in ecotoxicological risk assessment.^46^ Accurately predicting which metabolites are likely to form in fish and at what concentrations, however, remains a significant challenge, not least because of interspecies differences.

Fish’s metabolic capacity may be compromised by the limited supply of oxygen to their various organs and body tissues by virtue of their single circulatory system^138^ and ectothermic nature, resulting in a resting metabolic rate that is about ten times lower than in endotherms (such as mammals) of similar body mass.^204^ Moreover, both lower basal-level metabolic enzyme activities and slower clearance rates of environmental contaminants have been reported in fish compared to mammalian liver preparations.^205−208^ Temperature cycles play a major role in entraining the biological clocks that drive rhythmic physiological processes such as metabolism in fish,^209^ potentially giving rise to seasonal variations in drug plasma concentration and bioavailability. Indeed, metabolic rate (or enzyme activity) and elimination half-life in fish have been shown to correlate directly with ambient water and fish body temperature.^210−216^ Since metabolism is an enzyme-catalyzed process, this will most likely hold true up to the species’ evolutionary optimum temperature.^217^ Coldwater fish generally have lower metabolic rates than warm water species^167^ and, as such, may be less capable of metabolizing certain pharmaceuticals with the potential for a higher risk of experiencing toxicity. At the same time, lower environmental temperatures may also result in reduced pharmaceutical uptake rates and increased sequestration rates in lipid tissues. The ultimate effect of temperature on fish’s susceptibility to drug-induced toxicity will hence depend on whether uptake or detoxification processes have a greater temperature coefficient (Q10). Considering all the above, it cannot be assumed that all pharmaceuticals are metabolized via similar pathways and to similar extents in fish and in humans. Nevertheless, drugs that show low levels of hepatic clearance in humans or mammalian models should be prioritized for risk assessment in fish with due consideration given to coldwater species.

The composition and activity of the gut microbiota can have a major effect on pharmaceutical uptake, metabolism and toxicity as these microorganisms have the ability to activate prodrugs (e.g., sulfasalazine), deactivate APIs (e.g., digoxin) and convert drugs to toxic or reactive intermediates (e.g., NSAIDs).^218−220^ As such, it has been argued that interindividual variations in drug efficacy and toxicity are inextricably linked to variations in gut microflora. In fish, this array of microbes has been shown to be quite different from those seen in other vertebrates. While *Firmicutes* and *Bacteroidetes* are the predominant GIT bacterial phyla in most vertebrates,^221^ more than half of the average fish GIT microbial community is made up of *Proteobacteria*, with high proportions of *Firmicutes* (13.5%) and *Cyanobacteria* (10.3%) present as well.^222^ These significant compositional differences are bound to result in variations in drug biotransformation. Data regarding the specific mechanisms, responsible microbes and affected APIs are, however, still very limited. Additionally, environmental factors such as temperature and water salinity may modify the GIT microflora in fish,^222−224^ in turn affecting drug metabolism.

From the above analysis, a key message is that predicting drug metabolism and resultant concentrations and toxicities in fish based on mammalian data involves many uncertainties, thereby demanding a better understanding of pharmaceutical metabolism in fish to facilitate more accurate risk assessment.

### Excretion (E)

The kidney is regarded as the most important excretory organ for nitrogenous waste and drugs in humans and other mammals, with polar entities generally being more efficiently eliminated than those which are highly lipid soluble.^145^ The mesonephric kidney plays a minor role in nitrogenous waste excretion in most fish, but may have additional nonexcretory functions, such as hematopoiesis, not present in the mammalian metanephric kidney.^225^ Unlike mammals, the majority of fish species excrete nitrogenous waste as NH_3_ via the gills and skin, rather than storing or converting it to urea and uric acid.^100^ Despite limited data being available, the gills, skin, kidneys and liver (via the bile) all seem to be involved in pharmaceutical excretion in fish, the relative contribution of each route most likely depending on both fish- and drug-related factors, as seen for other xenobiotics. The polysaccharide laminaran, for example, was shown to be exclusively excreted in the urine of Atlantic cod (*Gadhus morhua*), while it concentrated in the bile of Arctic cod (*Boreogadus saida*), an aglomerular species.^226^ Branchial elimination, on the other hand, seems to be the most important excretory route for neutral, hydrophilic, low MW compounds such as aldicarb, an insecticide (log *K*_OW_ 1.13, MW 190.27), and 17α-methyltestosterone, an anabolic steroid (log *K*_OW_ 3.36, MW 302.5),^227−229^ although the excretion of hydrophobic compounds and ionizable drugs have also been noted across the rainbow trout gill *in vivo* and *in vitro*, respectively.^230,231^

Renal excretion generally involves three steps, namely glomerular filtration, tubular reabsorption and tubular secretion.^145,232^ During the first step, only unbound drug can be filtered. Hence, the anionic species of acids that tend to be highly bound to human plasma proteins often show lower clearance rates than bases.^62^ This might not be the case in fish, however, where some weak acids are less bound to plasma proteins (see Distribution). Aglomerular fish, of which more than 50 species have been identified,^233^ lack this passive filtration step and may exhibit slower total clearance rates as drug elimination will be limited to transporter-mediated excretion via the gills or bile.^226^ During the second step of renal excretion, substances are selectively, and often actively, removed from the filtrate (urine) and deposited back into the blood. For weak electrolytes, this step is pH-dependent:^145^ When the filtrate is more alkaline, weak acids are largely ionized and thus excreted more easily, but when it is more acidic, weak acids are less ionized and more easily reabsorbed, thus reducing their excretion. The opposite is true for weak bases. This effect is most pronounced for weak electrolytes with p*K*_a_ values in the range of urinary pH (5–8), which may differ among fish species. Marine fish, for example, have more acidic urine (Figure 2b) to limit the precipitation of calcium and magnesium during prolonged urine storage.^234^ The renal systems of these fish are thus better equipped for eliminating basic compounds, while acidic compounds may be subject to reabsorption, resulting in increased exposure and risk of toxicity. The final step of renal excretion involves the active carrier-mediated secretion of substances that were either too large to be filtered or are in excess from the peritubular capillary into the tubular fluid. During this step, P-gp, multidrug-resistance-associated proteins and solute carrier (SLC) transporters are responsible for the secretion of anionic drugs, conjugated metabolites and cationic drugs in humans, respectively.^145^ The contribution of active secretion to the renal excretion of xenobiotics and their metabolites has also been demonstrated in fish.^235^

Most fish possess a renal portal system that exposes the kidney tubules to a higher fraction of the cardiac output than in mammals.^210,227^ This system may subject a large portion of absorbed drug, especially from the skin,^236^ to a renal first-pass effect, leading to a significant reduction in bioavailability.^210^ Renal regeneration through *de novo* nephron neogenesis is another unique physiological feature that may potentially protect fish against drug-induced nephrotoxic harm.^237^ Given the contrasting composition of their habitats, FW and SW fish display some significant differences in terms of their renal systems.^238,239^ In FW fish, where osmosis promotes the absorption of water (Figure 2a), the kidneys are large in relation to the fish’s body weight, so as to maintain substantial glomerular filtration rates and excrete large volumes of urine, while simultaneously conserving essential ions via tubular reabsorption.^240^ SW fish, on the other hand, have relatively simple kidneys and only excrete very small volumes of concentrated urine (Figure 2b). Consequently, these fish are largely dependent on their gills for the excretion of nitrogenous waste, excess salts^239^ and, most probably, pharmaceuticals. It is interesting to note that euryhaline fish species can adjust their renal functions depending on the salinity of their environment.^238^ When migrating to freshwaters to spawn, these fish experience increased metabolic demands and a reduction in renal competence,^234^ potentially making them more susceptible to the effects of renally cleared pharmaceuticals during the spawning season.

Compounds that are excreted in human faeces mainly comprise orally ingested drugs that have not been absorbed or metabolites that have entered the GIT via active transporter-mediated secretions from bile or the bloodstream. An early study in rainbow trout found that parent compound and metabolite concentrations in bile can exceed those in the plasma and surrounding water,^241^ hence proving that fish also have the ability to bioconcentrate drugs in bile. Biliary excretion has indeed been shown to be important for the elimination of environmental contaminants generally in fish, particularly for chemicals that are highly polar and have MW > 600,^227^ but also for pharmaceuticals with relatively low MW, such as diclofenac (MW 296.1), carbamazepine (MW 236.27), fluoxetine (MW 309.33) and sertraline (MW 306.2).^242^ As for mammals, this process is most likely mediated by transporter proteins in fish.^98^ Noteworthy, biliary excretion rates in fish may vary with ambient temperature,^243^ such that the proportional excretion of pharmaceuticals in bile versus urine appears to be species-dependent.^244^

A notable carrier class involved in the excretion of endogenous metabolites and xenobiotics in mammals comprises the multidrug and toxin extrusion (MATE) proteins–bidirectional transporters belonging to the SLC superfamily. Although a number of SLCs have been identified in fish tissues, mammalian MATEs and fish Mates display both notable similarities and differences with respect to substrate specificity and affinity.^245^ Moreover, very few studies have focused on their expression levels and localization in fish. Six *mate* genes have been identified in the zebrafish genome, all of which were expressed in both adult and embryonic developmental stages.^245^ Expression levels were found to be the highest in the kidney and testes, followed by the liver and brain. A number of roles have subsequently been suggested for Mate proteins in fish, including the protection of early embryos against environmental toxins, the excretion of exogenous and endogenous compounds through the adult kidneys and liver, and the elimination of xenobiotics and/or metabolites via efflux into the intestinal lumen.^245^ With this information in mind, mammalian efflux transporter substrates potentially pose a high risk to fish as differences in the expression and specificity of transporter proteins may cause the rate and extent of pharmaceutical excretion to differ from those seen in mammals, thereby affecting drug accumulation, half-life and potential toxicity.

## Final Analysis

This critical review takes a detailed physiological perspective on the ERA of pharmaceuticals in fish. Fish are highly diverse, consisting of more than 30,000 extant species^71^ with distinctive physiological (and behavioral) attributes suited to a wide range of aquatic environmental conditions. Extrapolating mammalian data to predict pharmaceutical bioavailability and toxicity in fish is hence not a straightforward task and needs to be applied with caution. Optimizing the ERA process necessitates identifying potentially high-risk drug groups based on receiving environmental conditions, associated fish species and their physiological susceptibilities. Built on the current knowledge of pharmaceutical absorption, distribution, metabolism, excretion and toxicity (ADMET) in fish, a process-specific summary of the distinctive physiological features of fish expected to alter their susceptibility to pharmaceutical exposure and effects (compared to humans and mammalian models) is provided in the Supporting Information (Table S3, Supporting Information 1). Associated drug classes and priority fish groups/species for future research and risk assessments are also highlighted in this table. Among others, we identify that additional testing may be warranted for acidic APIs in general (see Distribution), highly hydrophilic and basic compounds in SW fish (see Absorption) as well as poorly metabolized drugs in coldwater species (see Metabolism). While considerable progress is being made in effects assessment by quantifying the levels of drug target conservation in increasing numbers of fish species, there is still a huge data gap in terms of the conservation of other proteins that drugs interact with, such as metabolic enzymes (e.g., CYPs) and their cofactors as well as drug PK and PD parameters in fish. Given the uncertainties in applying read-across from mammalian data, there is a need for fish-specific *in vitro* and/or *in silico* tools to help bridge this gap and to inform when *in vivo* testing in fish is likely to be necessary. Furthermore, future research strategies should focus on gaining more in-depth knowledge about ADME-related attributes that make fish more or less susceptible to the effects of pharmaceuticals, how these attributes vary for different taxonomic groups and environments, and how they ultimately affect the fitness of individuals and populations.

## Supporting Information Available

Supporting Information 1: Key assumptions/limitations of the fish plasma model and how these may be addressed/refined (Table S1); Comparison of *in vivo* pharmaceutical metabolism in fish and humans (Table S2); Summary of API ADMET-related susceptibility attributes in fish, associated drug classes and priority fish groups/species (Table S3) (PDF)
Supporting Information 2: Detailed results from online ortholog prediction tools used to construct Figure 3 (XLSX)

## Notes

This study did not generate any new data.

## Abbreviations

ABC: ATP-binding cassette
ADMET: absorption, distribution, metabolism, excretion and toxicity
AGP: α1-acid glycoprotein
API: active pharmaceutical ingredient
BCF: bioconcentration factor
CO_2_: carbon dioxide
CYP: cytochrome P450 enzyme
ER: effect ratio
ERA: environmental risk assessment
FDA: United States Food and Drug Administration
FPM: fish plasma model
F_SS_PC: fish steady-state plasma concentration
FW: freshwater
GIT: gastrointestinal tract
HSA: human serum albumin
H_T_PC: human therapeutic plasma concentration
log *D*: pH-dependent distribution coefficient
log *K*_OW_: *n*-octanol/water partition coefficient
MATE: multidrug and toxin extrusion protein
MOA: mode of action
MW: molecular weight
NH_3_: ammonia
NSAID: nonsteroidal anti-inflammatory drug
PD: pharmacodynamics
P-gp: P-glycoprotein
PK: pharmacokinetics
p*K*_a_: acid dissociation constant
PPB: plasma protein binding
PXR: pregnane X receptor
SLC: solute carrier
SW: saltwater
WWTP: wastewater treatment plant
