# Correction to “Factors Determining the Susceptibility of Fish to Effects of Human Pharmaceuticals”

**DOI:** 10.1021/acs.est.3c07375

**Published:** 2023-09-21

**Authors:** Chrisna Matthee, Andrew Ross Brown, Anke Lange, Charles R. Tyler

In the abstract, fish should have been correctly characterized as being ectothermic.

