# Supplementary material for: Factors Determining the Susceptibility of Fish to Effects of Human Pharmaceuticals
Source: Environ Sci Technol. 2023 Jun 8;57(24):8845–62. doi: 10.1021/acs.est.2c09576 (PMC10286317; doi:10.1021/acs.est.2c09576)
Supplement: Supplementary file 1 — es2c09576_si_001.pdf [file es2c09576_si_001.pdf]

Factors Determining the Susceptibility of Fish to Effects of Human Pharmaceuticals

Chrisna Matthee, Andrew Ross Brown, Anke Lange and Charles R Tyler

Biosciences, University of Exeter, Exeter, Devon, EX4 4QD, UK

Supporting Information 1

Table of Content

Table S1. Key assumptions/limitations of the Fish Plasma Model and how these may be addressed/refined

Table S2. Comparison of *in vivo* pharmaceutical metabolism in fish and humans

Table S3. Summary of API ADMET-related susceptibility attributes in fish, associated drug classes and priority fish groups/species

References

Table S1. Key assumptions/limitations of the Fish Plasma Model and how these may be addressed/refined

ASSUMPTION/LIMITATION	SUGGESTED REFINEMENTS	DATA REQUIREMENTS
API uptake is assumed to be a passive bioconcentration process[1]	Consider active uptake of APIs	Active uptake of APIs in fish<br>How these may contribute to the exposure and effects of specific APIs
Uptake via water is assumed to be the primary route of exposure[2]	Consider all uptake routes (i.e., branchial, dermal and dietary)	How environmental factors, drug properties and fish physiology affect the relative contribution of each uptake route
Internal and external aqueous concentrations are assumed to be equal or closely related[1,3]	Consider ADME processes when estimating internal concentrations.<br>Base $F_{SS}PC$ predictions on BCFs,[3] measured whole-body tissue concentrations, or a combination of simple QSARs and water exposure concentrations[2]	How tissue and body fluid concentrations relate to external aqueous concentration<br>How these are affected by environmental factors, drug properties and fish physiology
Molecular targets are assumed to be common, and their activation is assumed to lead to similar effects in humans and fish[1-4]<br>API potency is assumed to be similar between fish and mammals[5]	Consider differences in target sensitivity and functionality between mammals and fish, as well as across different fish species (e.g., using a functional equivalence ratio)[5]<br>Consider the potential interaction of APIs with off-targets in fish	Conservation of specific human drug targets, including their sensitivity and functionality in fish<br>The role(s) of specific drug targets in normal fish physiology[6]<br>The potency of specific APIs in different fish species<br>Potential off-targets of specific APIs in fish
H_TPC is assumed to equate to adverse physiological concentrations in fish (i.e., no adverse effects below H_TPC)[1-4,7]	Employ appropriate safety factors and refine them as new data becomes available	NOEC and/or LOEC values needed for specific APIs and fish species and/or life stages
Plasma composition is assumed to be similar in fish and mammals	Consider differences in plasma protein and lipid content between fish and humans	Plasma compositions of different fish species
Drug binding to plasma components is assumed to be similar across species and to be primarily driven by hydrophobicity[1]	Consider differences in drug binding to plasma components between humans and fish	Bound versus unbound fractions of specific APIs in fish plasma

The importance of API metabolism is neglected and/or assumed to be similar across species[1]	Consider factors affecting the rate and extent of API metabolism (i.e., activity level, temperature, life stage, CYP expression, etc.)	How API metabolism differs between fish and humans, as well as between different fish species<br>Expression, activity and substrate specificity of specific drug-metabolising enzymes
The parent compound is assumed to be solely responsible for effects/toxicity; (re)active metabolites are often ignored	Consider the extent of parent compound metabolism and resultant metabolite concentration(s)<br>Consider the contribution of metabolites to effects/toxicity	Formation of (re)active metabolites for specific APIs in fish<br>Potential effects/toxicity of these metabolites in fish
Mainly applicable to hydrophobic, non-ionising compounds, with K_{OW} being used to predict lipophilicity and fish plasma concentrations[4,8]	Account for ionisation and the effect of pH by using experimentally determined log D instead of log K_{OW} values	Experimental log D values needed for high-risk APIs in fish
Fish life stage (and associated physiological changes) are not taken into account	Consider the potential impact of fish life stage-related differences on drug ADMET	Influence of life stage on ADMET
Only focuses on API's primary target and/or MOA[6]	Consider potential off-targets and secondary MOAs	Off-targets and secondary MOAs of high-risk APIs in fish
Biotransformation by gut microbiota not considered	Consider the potential metabolism of drugs and formation of (re)active metabolites in the gut	Type of metabolic reactions that may occur in the fish gut<br>Drugs that are (or are likely to be) involved in these reactions
Season- and species-specific lipid turnover dynamics are not considered.	Consider potential effect of unique lipid storage strategies on API distribution, storage and metabolism	Relevant fish species, APIs and likely effects
Environmental conditions (SW vs FW; water temperature) are not taken into account	Consider potential effects of environmental factors (specifically water salinity and temperature) on ADMET	Extent to which different environmental conditions may affect drug ADMET
Unique fish behaviours and/or physiologies are not taken into account	Consider the potential effects of unique fish behaviours (i.e., anadromous species) and physiologies (i.e., scaleless or aglomerular species) on ADMET	Extent to which unique behaviours/physiologies may affect drug ADMET

Abbreviations: ADMET, absorption, distribution, metabolism, excretion and toxicity; API, active pharmaceutical ingredient; BCF, bioconcentration factor; C_{max}, maximum plasma concentration; CYP, cytochrome P450; F_{SS}PC, fish steady state plasma concentration; FW, freshwater; H_TPC, human therapeutic plasma concentration; LOEC, lowest observed effect concentration; log D, distribution coefficient; log K_{OW}, n-octanol/water partition coefficient; MOA, mechanism of action; NOEC, no observed effect concentration; PPB, plasma protein binding; QSAR, quantitative structure-activity relationship; SW, saltwater.

Table S2. Comparison of *in vivo* pharmaceutical metabolism in fish and humans

Active Pharmaceutical Ingredient	Fish Species	Human Metabolism			Fish Metabolism	
		Metabolites	Pathways	Enzymes	Metabolites	Pathways*
Albendazole (ABZ)[9-11]	Rainbow trout (*Oncorhynchus mykiss*)<br>Tilapia hybrid (*Oreochromis nilotica x O. mosambicus*)<br>Atlantic salmon (*Salmo salar*)	ABZ sulfoxide (major, active)<br>ABZ sulfone<br>ABZ aminosulfone	Oxidation<br>Deacetylation	FMO<br>CYP3A	All three major metabolites identified in all three fish species.<br>*Hydroxy-ABZ?*	Oxidation<br>Deacetylation<br>*Hydroxylation?*
Beclomethasone dipropionate[12-14]	Rainbow trout (*Oncorhynchus mykiss*)	Beclomethasone 17-monopropionate (major, active)<br>Beclomethasone 21-monopropionate (minor, inactive)<br>Beclomethasone (minor, inactive)	Hydrolysis	Esterases	Beclomethasone 17-monopropionate<br>Beclomethasone	Hydrolysis
	Fathead minnow (*Pimephales promelas*)				Beclomethasone 17-monopropionate<br>Beclomethasone	Hydrolysis
Carbamazepine (CBZ)[15-17]	Killifish (*Jenynsia multidentata*)	CBZ 10,11-epoxide (major, active)<br>Hydroxy metabolites<br>Quinone metabolites<br>Conjugated metabolites	Oxidation<br>Hydroxylation<br>Hydrolysis<br>Conjugation	CYP3A4<br>CYP2C8<br>Epoxide hydrolase<br>β-glucuronidase	CBZ 10,11-epoxide<br>2-hydroxy CBZ<br>(no other metabolites investigated)	Oxidation<br>Hydroxylation
Chlorpromazine (CPZ)[18-20]	Zebrafish (*Danio rerio*) larvae	7-hydroxy CPZ (active)<br>3-hydroxy CPZ	Oxidation<br>Dealkylation	CYP2D6<br>CYP1A2	Mono-oxidised metabolites<br>Di-oxidised metabolites	Oxidation<br>Demethylation

		CPZ N-oxide<br>De-monomethyl CPZ<br>De-dimethyl CPZ<br>Conjugated metabolites<br>Other minor metabolites	Demethylation<br>Hydroxylation<br>Conjugation	CYP3A4	De-monomethyl CPZ	
Cisapride[19,21,22]	Zebrafish (*Danio rerio*) larvae	Norcisapride (major)<br>3-fluoro-4-hydroxycisapride<br>4-fluoro-2-hydroxycisapride<br>Glucuronide conjugates<br>Other minor metabolites	Dealkylation<br>Aromatic hydroxylation<br>Oxidation<br>Conjugation	CYP3A4 (mainly)<br>CYP2A6	Cisapride N-sulfate<br>(no major human metabolites observed)	Sulphate conjugation
Dextromethorphan[19,23]	Zebrafish (*Danio rerio*) larvae	Dextrorphan<br>3-methoxymorphinan<br>3-hydroxymorphinan<br>Conjugated metabolites	Demethylation<br>Hydroxylation<br>Conjugation	CYP2D6<br>3A4<br>UGTs	Dextrorphan<br>3-methoxymorphinan<br>Hydroxyl metabolite	Demethylation<br>Hydroxylation
Diltiazem (DTZ)[24,25]	Rainbow trout (*Oncorhynchus mykiss*)	N-monodesmethyl DTZ (active)<br>Deacetyl DTZ (active)<br>Deacetyl N-monodesmethyl DTZ (active)<br>Other minor metabolites	Demethylation<br>Deacetylation<br>Oxidation<br>Oxidative deamination<br>Conjugation	CYP3A4<br>CYP2D6<br>CYP2C8<br>CYP2C9<br>Esterases	N-monodesmethyl DTZ<br>Deacetyl DTZ<br>Deacetyl N-monodesmethyl DTZ<br>Hydroxyl DTZ (fish-specific)<br>Other minor metabolites	Demethylation<br>Deacetylation<br>Hydroxylation (fish-specific)
Fluoxetine[26,27]	Japanese medaka (*Oryzias latipes*)	Norfluoxetine (major, active)<br>Glucuronides<br>Other minor metabolites	Demethylation<br>Conjugation	CYP2D6<br>CYP2C9<br>CYP3A4/5<br>CYP2C19	Norfluoxetine<br>(no other metabolites investigated)	Demethylation

Selegiline[28-30]	Zebrafish (*Danio rerio*)	(R)-methamphetamine<br>(R)-amphetamine<br>(R)-desmethylselegiline<br>(1S,2R)-norephedrine<br>(1R,2R)-norpseudoephedrine<br>(1S,2R)-ephedrine<br>(1R,2R)-psuedoephedrine<br>(R)-p-hydroxyamphetamine<br>(R)-p-hydroxymethamphetamine	N-dealkylation<br>β-carbon hydroxylation<br>Ring-hydroxylation	CYP2B6<br>CYP2C19<br>CYP3A4<br>CYP1A2	(R)-methamphetamine(R)-amphetamine<br>(R)-desmethylselegiline<br>(R)-p-hydroxyamphetamine<br>(R)-p-hydroxymethamphetamine	N-dealkylation<br>Ring-hydroxylation<br>(no β-carbon hydroxylation)
Tramadol[31-34]	Fathead minnow (*Pimephales promelas*)	O-desmethyl tramadol (major, active)<br>N-desmethyl tramadol (major, inactive)<br>O,N-didesmethyl tramadol<br>N,N-didesmethyl tramadol<br>N,N,O-tridesmethyl tramadol<br>Conjugates<br>Other minor metabolites	Demethylation (major)<br>Oxidation (major)<br>Oxidative N-dealkylation<br>Dehydration<br>Conjugation	CYP2D6<br>CYP3A4<br>CYP2B6	O-desmethyl tramadol<br>N-desmethyl tramadol<br>(no other metabolites investigated)	Demethylation
Verapamil[19,27]	Zebrafish (*Danio rerio*) larvae	D-617 (major)<br>Norverapamil (active)<br>Other minor metabolites: D-702, D-703, D-620, D-715, D-717	N-dealkylation<br>Demethylation	CYP3A4<br>CYP3A5<br>CYP2C8	D-617<br>D-620<br>*Norverapamil?*<br>Several fish-specific metabolites	Oxidation<br>Demethylation<br>Glucuronide conjugation<br>N-dealkylation

*Fish metabolic pathways were assumed based on detected metabolites. Abbreviations: ABZ, albendazole; CBZ, carbamazepine; CPZ, chlorpromazine; CYP, cytochrome P450; DTZ, diltiazem; FMO, flavin-containing monooxygenase; UGT, uridine 5'-diphospho-glucuronosyltransferase.

Table S3. Summary of API ADMET-related susceptibility attributes in fish, associated drug classes and priority fish groups/species

	ATTRIBUTES	DRUG CLASSES	PRIORITY FISH GROUPS/SPECIES
TOXICITY (T)	High human drug target conservation	• Multi-target compounds	-
	Off-targets and/or homologues	• Compound classes with conserved MOAs and/or additive effects	-
	Role of innate immune system	Immunomodulatory drugs	-
ABSORPTION (A)	Multiple uptake routes	-	-
	Variable composition and structure of biological membranes	-	-
	Highly specialised, multifunctional gills	• Compounds using both passive and active uptake mechanisms<br>• Ionisable compounds<br>• Low MW compounds<br>• Compounds with log K_{OW} 3-6	• Highly active species<br>• Species living in acidic environments<br>• FW species
	Varied membrane transporter expression and substrate specificities	Human drug transporter substrates	-
	Varied water consumption, gut epithelium permeability and intestinal fluid pH	Hydrophilic compounds<br>Orally active compounds<br>Basic compounds	• SW/marine species<br>• Anadromous and euryhaline species
	Constant skin-water contact and non-keratinised epidermis	Neutral compounds<br>Sediment-associated compounds	• Embryo-larval stages<br>• Juveniles<br>• Some small-sized species<br>• Scaleless species<br>• Demersal/benthic species<br>• FW species (theoretically)
DISTRIBUTION (D)	Single circulatory circuit	-	-
	Varied PPB due to…<br>• Low plasma protein content (compared to humans);<br>• Varied plasma protein structure and function	• Highly (human) protein-bound compounds<br>• Weak acids<br>• Compounds with low log K_{OW}<br>• Narrow TI compounds	-

	• High levels of apolipoproteins in plasma		
	High blood pH (compared to humans)	Weak acids and bases (pKa 5-9)	-
	Seasonal lipid storage strategies	Lipophilic compounds	• Anadromous species<br>• Embryo-larval stages
	Varied efflux transporter expression and substrate specificities	• Human P-gp substrates<br>• Chemo-sensitisers	-
**METABOLISM (M)**	Varied metabolic enzyme expression, activity and substrate specificities	• Human CYP substrates, inducers & inhibitors<br>• Compounds with promiscuous functional groups and/or (re)active metabolites	-
	Limited metabolic capacity due to single blood flow circuit & ectothermic nature	Poorly metabolised compounds	Coldwater species
	Unique gut microbiota composition	Compounds transformed by gut microbes	-
**EXCRETION (E)**	Varied contribution of kidneys, gills (major), skin and liver to xenobiotic excretion	-	-
	Varied renal system structure (i.e., presence/absence of glomerulus)	(Human) glomerular filtered compounds	Aglomerular species
	Varied urine pH	Weak acids	SW/marine species
	Adjustable renal functioning	(Human) renally cleared compounds	Anadromous and euryhaline species
	Varied efflux transporter expression and substrate specificities	Human efflux transporter substrates	-

Empty cells indicate that information is not known or is not applicable. Abbreviations: CYP, cytochrome P450 enzymes; FW, freshwater; K_{OW}, n-octanol/water partition coefficient; MOA, mechanism of action; MW, molecular weight; P-gp, P-glycoprotein; PPB, plasma protein binding; SW, saltwater; TI, therapeutic index.

.

References

(1) Henneberger, L.; Kluver, N.; Muhlenbrink, M.; Escher, B. Trout and human plasma protein binding of selected pharmaceuticals informs the Fish Plasma Model. *Environmental Toxicology and Chemistry* **2020**, 1-10. DOI: 10.1002/etc.4934.

(2) Meador, J. P.; Yeh, A.; Gallagher, E. P. Determining potential adverse effects in marine fish exposed to pharmaceuticals and personal care products with the fish plasma model and whole-body tissue concentrations. *Environmental Pollution* **2017**, *230,* 1018-1029. DOI: http://dx.doi.org/10.1016/j.envpol.2017.07.047.

(3) Schreiber, R.; Gündel, U.; Franz, S.; Küster, A.; Rechenberg, B.; Altenburger, R. Using the fish plasma model for comparative hazard identification for pharmaceuticals in the environment by extrapolation from human therapeutic data. *Regulatory Toxicology and Pharmacology* **2011**, *61,* 261-275. DOI: 10.1016/j.yrtph.2011.08.006.

(4) Weil, M.; Falkenhain, A.; Scheurer, M.; Ryan, J. J.; Coors, A. Uptake and effects of beta-adrenergic agonist salbutamol in fish: supporting evidence for the fish plasma model. *Environmental Toxicology and Chemistry* **2019**, *38* (11), 2509-2519. DOI: 10.1002/etc.4543.

(5) Owen, S. F.; Giltrow, E.; Huggett, D. B.; Hutchinson, T. H.; Saye, J.; Winter, M. J.; Sumpter, J. P. Comparative physiology, pharmacology and toxicology of β-blockers: mammals versus fish. *Aquatic Toxicology* **2007**, *82,* 145-162. DOI: 10.1016/j.aquatox.2007.02.007.

(6) Rand-Weaver, M.; Margiotta-Casaluci, L.; Patel, A.; Panter, G. H.; Owen, S. F.; Sumpter, J. P. The read-across hypothesis and environmental risk assessment of pharmaceuticals. *Environmental Science & Technology* **2013**, *47,* 11384-11395. DOI: dx.doi.org/10.1021/es402065a.

(7) Huggett, D. B.; Cook, J. C.; Ericson, J. F.; Williams, R. T. A theoretical model for utilizing mammalian pharmacology and safety data to prioritize potential impacts of human pharmaceuticals to fish. *Human and Ecological Risk Assessment* **2003**, *9* (7), 1789-1799. DOI: 10.1080/10807030390260498.

(8) Bao, Y.; Huang, W.; Hu, X.; Yin, D. Distribution of 31 endocrine-disrupting compounds in the Taihu Lake and application of the fish plasma model. *Environmental Sciences Europe* **2020**, *32,* 1-16. DOI: https://doi.org/10.1186/s12302-020-00347-0.

(9) Shaikh, B.; Rummel, N.; Gieseker, C.; Serfling, S.; Reimschuessel, R. Metabolism and residue depletion of albendazole and its metabolites in rainbow trout, tilapia and Atlantic salmon after oral administration. *Journal of Veterinary Pharmacology and Therapeutics* **2003**, *26* (6), 421-427. DOI: 10.1046/j.0140-7783.2003.00534.x

(10) Mirfazaelian, A.; Dadashzadeh, S.; Rouini, M. R. Effect of gender in the disposition of albendazole metabolites in humans. *European Journal of Clinical Pharmacology* **2002**, *58,* 403-408. DOI: 10.1007/s00228-002-0488-8.

(11) McCarthy, J. S.; Moore, T. A. Drugs for Helminths. In *Mandell, Douglas, and Bennett's Principles and Practice of Infectious Diseases,* 8th ed.; Bennett, J. E., Dolin, R., Blaser, M. J. Eds.; Vol. 1; Elsevier, **2015**; p 519.

(12) Daley-Yates, P. T.; Price, A. C.; Sisson, J. R.; Pereira, A.; Dallow, N. Beclomethasone dipropionate: absolute bioavailability, pharmacokinetics and metabolism following intravenous, oral, intranasal and inhaled administration in man. *British Journal of Clinical Pharmacology* **2001**, *51* (5), 400-409. DOI: 10.1046/j.0306-5251.2001.01374.x

(13) Almroth, B. M. C.; Gunnarsson, L. M.; Cuklev, F.; Fick, J.; Kristiansson, E.; Larsson, D. G. J. Waterborne beclomethasone dipropionate affects the physiology of fish while its metabolite beclomethasone is not taken up. *Science of the Total Environment* **2015**, *511*, 37-46. DOI: http://dx.doi.org/10.1016/j.scitotenv.2014.12.016.

(14) Margiotta-Casaluci, L.; Owen, S.; Huerta, B.; Rodriguez-Mozaz, S.; Kugathas, S.; Barceló, D.; Rand-Weaver, M.; Sumpter, J. P. Internal exposure dynamics drive the Adverse Outcome Pathways of synthetic glucocorticoids in fish. *Scientific Reports* **2016**, *6*, 21978. DOI: https://doi.org/10.1038/srep21978.

(15) Valdés, M. E.; Huerta, B.; Wunderlin, D. A.; Bistoni, M. A.; Barcelo, D.; Rodriguez-Mozaz, S. Bioaccumulation and bioconcentration of carbamazepine and other pharmaceuticals in fish under field and controlled laboratory experiments. Evidences of carbamazepine metabolization by fish. *Science of the Total Environment* **2016**, *557-558*, 58-67. DOI: http://dx.doi.org/10.1016/j.scitotenv.2016.03.045.

(16) Cunningham, V. L.; Perino, C.; D'Aco, V. J.; Hartmann, A.; Bechter, R. Human health risk assessment of carbamazepine in surface waters of North America and Europe. *Regulatory Toxicology and Pharmacology* **2010**, *56*, 343-351. DOI: 10.1016/j.yrtph.2009.10.006.

(17) Breton, H.; Cociglio, M.; Bressolle, F.; Peyriere, H.; Blayac, J. P.; Hillaire-Buys, D. Liquid chromatography-electrospray mass spectrometry determination of carbamazepine, oxcarbazepine and eight of their metabolites in human plasma. *Journal of Chromatography* **2005**, *828*, 80-90. DOI: 10.1016/j.jchromb.2005.09.019.

(18) *DrugBank Online*. https://go.drugbank.com/ (accessed 2023-04-20).

(19) Alderton, W.; Berghmans, S.; Butler, P.; Chassaing, H.; Fleming, A.; Golder, Z.; Richards, F.; Gardner, I. Accumulation and metabolism of drugs and CYP probe substrates in zebrafish larvae. *Xenobiotica* **2010**, *40* (8), 547-557. DOI: 10.3109/00498254.2010.493960.

(20) Green, D. E.; Forrest, I. S. *In vivo* metabolism of chlorpromazine. *Canadian Psychiatric Association Journal* **1966**, *11* (4), 299-302.

(21) Bohets, H.; Lavrijsen, K.; Hendrickx, J.; van Houdt, J.; van Genechten, V.; Verboven, P.; Meuldermans, W.; Heykants, J. Identification of the cytochrome P450 enzymes involved in the metabolism of cisapride: *in vitro* studies of potential co-medication interactions. *British Journal of Pharmacology* **2000**, *129*, 1655-1667.

(22) Meuldermans, W.; Van Peer, A.; Hendrickx, J.; Lauwers, W.; Swysen, E.; Bockx, M.; Woestenborghs, R.; Heykants, J. Excretion and biotransformation of cisapride in dogs and humans after oral administration. *Drug Metabolism and Disposition* **1988**, *16* (3), 403-409.

(23) Taylor, C. P.; Traynelis, S. F.; Siffert, J.; Pope, L. E.; Matsumoto, R. R. Pharmacology of dextromethorphan: relevance to dextromethorphan/quinidine (Nuedexta®) clinical use. *Pharmacology & Therapeutics* **2016**, *164*, 170-182. DOI: https://doi.org/10.1016/j.pharmthera.2016.04.010.

(24) Yeung, P. K. F.; Prescott, C.; Haddad, C.; Montague, T. J.; McGregor, C.; Quilliam, M. A.; Xei, M.; Farmer, R. L.; Klassen, G. A. Pharmacokinetics and metabolism of diltiazem in healthy males and females following a single oral dose. *European Journal of Drug Metabolism and Pharmacokinetics* **1993**, *18*, 199-206.

(25) Steinbach, C.; Grabic, R.; Fedorova, G.; Koba, O.; Golovko, O.; Grabicova, K.; Kroupova, H. K. Bioconcentration, metabolism and half-life time of the human therapeutic drug diltiazem in rainbow trout *Oncorhynchus mykiss*. *Chemosphere* **2016**, *144*, 154-159. DOI: http://dx.doi.org/10.1016/j.chemosphere.2015.08.038.

(26) Paterson, G.; Metcalfe, C. D. Uptake and depuration of the anti-depressant fluoxetine by the Japanese medaka (*Oryzias latipes*) *Chemosphere* **2008**, *74* (1), 125-130. DOI: 10.1016/j.chemosphere.2008.08.022

(27) Whirl-Carrillo, M.; Huddart, R.; Gong, L.; Sangkuhl, K.; Thorn, C. F.; Whaley, R.; Klein, T. E. An evidence-based framework for evaluating pharmacogenomics knowledge for personalized medicine. *Clinical Pharmacology and Therapeutics* **2021**, *110* (3), 563–572. DOI: https://doi.org/10.1002/cpt.2350.

(28) Sardela, V. F.; Anselmo, C. S.; Nunes, I. K. D. C.; Carneiro, G. R. A.; Dos Santos, G. R. C.; de Carvalho, A. R.; Labanca, B. J.; Silva Oliveira, D.; Ribeiro, W. D.; de Araujo, A. L. D.; Padilha, M. C.; de Lima, C. K. F.; de Sousa, V. P.; de Aquino Neto, F. R.; Gualberto Pereira, H. M. Zebrafish (*Danio rerio*) water tank model for the investigation of drug metabolism: Progress, outlook, and challenges. *Drug Testing and Analysis* **2018**, *10* (11-12), 1657–1669. DOI: https://doi.org/10.1002/dta.2523.

(29) Shin, H. S. Metabolism of selegiline in humans. Identification, excretion, and stereochemistry of urine metabolites *Drug Metabolism and Disposition* **1997**, *25* (6), 657-662.

(30) Hidestrand, M.; Oscarson, M.; Salonen, J. S.; Nyman, L.; Pelkonen, O.; Turpeinen, M.; Ingelman-Sundberg, M. CYP2B6 and CYP2C19 as the major enzymes responsible for the metabolism of selegiline, a drug used in the treatment of Parkinson's disease, as revealed from experiments with recombinant enzymes. *Drug Metabolism and Disposition* **2001**, *29* (11), 1480-1484.

(31) Vazzana, M.; Andreani, T.; Fangueiro, J.; Faggio, C.; Silva, C.; Santini, A.; Garcia, M. L.; Silva, A. M.; Souto, E. B. Tramadol hydrochloride: Pharmacokinetics, pharmacodynamics, adverse side effects, co-administration of drugs and new drug delivery systems. *Biomedicine & Pharmacotherapy* **2015**, *70*, 234-238. DOI: http://dx.doi.org/10.1016/j.biopha.2015.01.022.

(32) Tanoue, R.; Margiotta-Casaluci, L.; Huerta, B.; Runnalls, T. J.; Nomiyama, K.; Kunisue, T.; Tanabe, S.; Sumpter, J. P. Uptake and metabolism of human pharmaceuticals by fish: a case study with the opioid analgesic tramadol. *Environmental Science & Technology* **2017**, *51*, 12825-12835. DOI: 10.1021/acs.est.7b03441.

(33) Grond, S.; Sablotzki, A. Clinical pharmacology of tramadol. *Clinical Pharmacokinetics* **2004**, *43*, 879-923. (acccessed 17 Apr 2023).Gale Academic OneFile.

(34) Wu, W. N.; McKown, L. A.; Liao, S. Metabolism of the analgesic drug ULTRAM® (tramadol hydrochloride) in humans: API-MS and MS/MS characterization of metabolites. *Xenobiotica* **2002**, *32* (5), 411-425. DOI: 10.1080/00498250110113230.
